# Efficiency of a French-language triage algorithm in the Emergency Department

**DOI:** 10.1186/cc9875

**Published:** 2011-03-11

**Authors:** J Jobé, A Ghuysen, P Gérard, V D'Orio

**Affiliations:** 1 University Hospital, Liège, Belgium; 2 University, Liège, Belgium

## Introduction

ELISA (Echelle Liégeoise d'Index de Sévérité à l'Admission) is a valid new Emergency Department (ED) triage algorithm including five levels of categorisation (from U1, high emergency degree, to U5, low degree), based on vital signs and selected anamnestic data. Previous work has demonstrated that ELISA evidenced a strong inter-rater and intra-rater agreement [1]. In this study, we aimed at further evaluating its efficiency.

## Methods

From March 2008 to May 2008, we prospectively investigated 545 consecutive admissions to study the potential correlation between ELISA score and impact on resource consumption as well as ED stay. Resources were classified following three categories: complementary examinations (ECG, blood analysis, X-ray, and so forth), medical treatments (i.v. medications, casts, sutures, and so forth), and outcome after ED admission (discharge, hospitalisation, ICU admission or death). Each resource was considered a binary variable and was analyzed owing to four statistical tests: chi-square, Wald Wolfowitz, Kolmogorov- Smirnov and Mann-Whitney.

## Results

Statistical analysis evidenced an effect of ELISA score on the overall need for complementary examinations except for serology, X-rays and Holter ECG. The initial index severity had also related the need for urgent treatments. Outcomes were also significantly correlated with ELISA: the smaller the index, the bigger the number of hospitalisations, ICU admissions and deaths. This study demonstrates ELISA's efficiency; when the initial severity index is close to U1, more complementary examinations are needed and more medical treatments are necessary. Wounds do not have a high emergency degree, which explains why there was no influence of initial index severity on the realisation of sutures. The same reasoning is applied to X-rays that are frequently requested for light traumatic cases with low emergency degree. Finally, hospitalisation, ICU admission and death are more frequent when the ELISA score evidences the highest emergency degree.

## Conclusions

In addition to previous work demonstrating a strong inter-rater and intra-rater agreement, the present study points out the potent efficiency of ELISA, allowing its further use in the ED.
